# Potential Effects of Horizontal Gene Exchange in the Human Gut

**DOI:** 10.3389/fimmu.2017.01630

**Published:** 2017-11-27

**Authors:** Aaron Lerner, Torsten Matthias, Rustam Aminov

**Affiliations:** ^1^B. Rappaport School of Medicine, Technion-Israel Institute of Technology, Haifa, Israel; ^2^AESKU.KIPP Institute, Wendelsheim, Germany; ^3^Institute of Fundamental Medicine and Biology, Kazan Federal University, Kazan, Russia; ^4^School of Medicine & Dentistry, University of Aberdeen, Aberdeen, United Kingdom

**Keywords:** probiotics, microbiome, dysbiome, horizontal gene transfer, intestine, gut, biofilm, environment

## Abstract

Many essential functions of the human body are dependent on the symbiotic microbiota, which is present at especially high numbers and diversity in the gut. This intricate host–microbe relationship is a result of the long-term coevolution between the two. While the inheritance of mutational changes in the host evolution is almost exclusively vertical, the main mechanism of bacterial evolution is horizontal gene exchange. The gut conditions, with stable temperature, continuous food supply, constant physicochemical conditions, extremely high concentration of microbial cells and phages, and plenty of opportunities for conjugation on the surfaces of food particles and host tissues, represent one of the most favorable ecological niches for horizontal gene exchange. Thus, the gut microbial system genetically is very dynamic and capable of rapid response, at the genetic level, to selection, for example, by antibiotics. There are many other factors to which the microbiota may dynamically respond including lifestyle, therapy, diet, refined food, food additives, consumption of pre- and probiotics, and many others. The impact of the changing selective pressures on gut microbiota, however, is poorly understood. Presumably, the gut microbiome responds to these changes by genetic restructuring of gut populations, driven mainly *via* horizontal gene exchange. Thus, our main goal is to reveal the role played by horizontal gene exchange in the changing landscape of the gastrointestinal microbiome and potential effect of these changes on human health in general and autoimmune diseases in particular.

## Introduction

Single-celled microorganisms have shaped our planet for several billions of years before the arrival of multicellular organisms. The appearance of the humankind is a relatively recent evolutionary event but, within a very short time on the evolutionary scale, it has become a very powerful force on the planetary scale. It extensively changes the biosphere, consumes copious natural resources, affects global climate, and engages in wars that are driven by competition for natural resources, while the human population experiences an explosive growth, destroying natural ecosystems to sustain this growth. Interaction between humans and commensal microbiota shaped both of them, in a mutual and bidirectional way, thus benefiting microbes and us (1). Given the extensive influence of microorganisms across the entire biosphere, and the microbiota on human health, the gut–microbiome integrity is of prime significance to human health, disease prevention, and survival. In this regard, our body contains a “second genome,” allowing to cohabit with the human one to form a steady equilibrium for the two counterparts and for long-term mutual survival.

In parallel with the evolutionary selected adaptation mechanisms on both side of interaction, the new developments are entering this interaction to support and provide food for the ever-growing human population. These are the contemporary intensive agricultural systems with the widespread use of herbicides, insecticides, fungicides, fumigants, desiccants, harvest aids, antimicrobials, growth regulators, and many other substances. Other developments include the use of genetically manipulated microorganisms, plants, and animals as well as new nutrients, new food technologies, engineered microbial delivery systems, and various food additives.

Due to the close relationship and intimate cross talks between the human cells and gut microbiota, the effects of the latter on human health are substantial. Thus, the aim of the present manuscript is to update on potential outcomes of the microbiome, which is changing as a result of changing lifestyle, on human health in general and on chronic diseases in particular. It is not the aim of the present review to discuss horizontal gene transfer (HGT) in pathogens leading to the exchange of virulence genes that could contribute to the emergence of new “superbugs” (2). This review deals with the potential effects of our changing lifestyle on the evolutionary equilibrium with the “normal” microbiota, which had been established during the previous coevolution and cospeciation (3). The focus of this review is on horizontal gene exchange, which has been demonstrated for within the three domains of life, eukaryotes, bacteria, and archaea (4), with a particular emphasis on human host–microbe interaction. Finally, a potential role of HGT in the gut ecosystem in regard to human health and chronic disease induction, is discussed. While there are many other excellent reviews elsewhere discussing HGT in the gut, the main focus of these reviews is limited to the HGT events among the intestinal microbiota. In this review, broader implications of HGT are discussed that go beyond the gut microbiota boundaries and involve the host side as well.

## HGT: Definition, Characteristics, and Mechanisms

Horizontal gene transfer is the lateral exchange of genes between unicellular and/or multicellular organisms. In contrast to the vertical gene transfer, i.e., between generations, HGT enables the transfer of genetic sequences between remote species mediated usually by transformation, transduction, and conjugal transfer or with specific gene transfer agents (5, 6).

Horizontal gene transfer has been demonstrated for almost all phylogenetic groups in all three domains of life as a crucial factor in the evolution of various organisms (7–9), including plants (10, 11), viruses (12), archaea (13), fungi (14, 15), and animals (16). While evolution of the Eukaryotes is largely driven by vertical inheritance, the predominant form of evolution among the bacteria and archaea is HGT, the rate of which is comparable to the point mutation’s rate, surpassing the gene duplication rate (17). Thus, for these two domains, HGT is an essential way for genome diversification and novel function procurement to survive under the pressure of natural selection and to reproduce. Therefore, HGT is a main driver of microbial evolution and ecology. Bacteria have developed multiple natural genetic tools to exchange genetic material between strains, species, genera, and even higher taxa. While the phenomenon of HGT can be encountered in virtually any ecosystem, the focus of the present review is HGT in the human gut. This is complemented by a potential contribution of changing dietary and lifestyle habits to HGT.

One of the well-documented HGT events that the mankind has experienced in its history is the consequence of the rampant usage of antibiotics, which resulted in the widespread dissemination of antibiotic resistance genes among many bacteria (18). These mechanisms allowed them to acquire protection against the pressure of antibiotic selection to afford successful survival and proliferation. In general, HGT drives the enlargement of protein families in bacteria and archaea to deal with the environmental and anthropogenic impacts imposed upon them (19).

There are many mechanisms of HGT reviewed elsewhere (5, 6, 20–22). In brief, these mechanisms include the following.

**Transformation**: Genetic modification of the cell due to the uptake of foreign genetic material. Natural competence and transformation are relatively common in bacteria and, to some extent, in archaea, but not in eukaryotes. Artificial transformation is often used in recombinant technology for research, industrial or medical purposes.

**Transduction**: When microbial DNA is transferred from one bacterium to another one by a viral intermediate.

**Conjugation**: The transfer of DNA *via* a conjugative element [plasmid, transposon, ICE, or other mobile genetic elements (MGEs)] from a donor to a recipient cell during cell-to-cell touch (23, 24).

**Gene Transfer Agents**: Virus-like elements encoded by the host found in Alphaproteobacteria, order Rhodobacterales, and some other bacteria.

It is generally accepted that HGT *via* transduction, conjugation, and gene transfer agents is more efficient than transformation by naked DNA because in the former mechanisms DNA is protected from degradation. By means of HGT, entire genes and functional sequences can be incorporated into the genome of a recipient. Although the best-known examples are among the bacteria and archaea, they can be found in the Eukarya as well, including primates and humans (25). Thus, HGT had occurred and continues to occur, on a considerable scale, in all three domains of life, and is likely to be one of the important factors that have contributed to the diversification during evolution. Unlike evolution *via* gene duplications and mutations, which are slow and progressive processes, HGT allows a rapid acquisition of a new function important for species to pass through the natural selection barrier and successfully reproduce.

## HGT in the Gut

Since the gut is a niche mainly colonized by a plethora of microbial species, it is logical to presume that the genomes of methanogenic archaea in the intestine have acquired their ability to survive and proliferate in this environment through interdomain HGT from the microbial counterpart that dominate this niche. Recently, contribution of HGT to the gene repertoire in a gut-adapted commensal methanogen *Methanobrevibacter smithii*, which is the richest archaeon in the human gross intestine, has been evaluated (26). A phylogenetic tree-based genome-wide survey of putative genes, presumably acquired as a result of HGT, established that over 15% of the coding sequences in *M. smithii* could be inferred as of bacterial origin. Laterally acquired genes largely contribute to surface functions and encode glycosyltransferases and adhesin-like proteins, which also can act as virulence factors in pathogens. In addition, several important ABC transporters, especially metal transporters are potentially of microbial origin. Metals such as zinc, for example, are important for bacterial growth, and there is a strong competition for it among intestinal microbiota as well (27). Thus, the microbial genes acquired by this archaeon contributed to the host adaptation by permitting an extended variety of surface structures and enhancing the efficiency of metal ion uptake in the competitive gut niche. Taken together, adaptation of *M. smithii* to the niche involved the acquisition of bacterial genes into its genome to adjust its lifestyle.

A comparative study of fecal samples from mono and dizygotic twins revealed that the pan-genome of *M. smithii* “contains 987 genes conserved in all strains, and 1,860 variably represented genes” (28). Strains from monozygotic and dizygotic twins had a comparable degree of shared genes and SNPs and were significantly more similar than strains isolated from mothers or members of their families. The 101 adhesin-like proteins in the pan-genome (45 ± 6 per strain) exhibited strain-specific differences in expression and responsiveness to format. The authors hypothesized that *M. smithii* strains use their different repertoires of adhesin-like proteins to create diversity in their metabolic niches, by permitting them to create syntrophic relationships with bacterial partners with differential metabolic capacities and patterns of co-occurrence. It is generally accepted that the core genome genes are less prone to HGT than that of the auxiliary genome. Thus, the majority of genes in the pan-genome of *M. smithii* are laterally circulating among the strains of this species.

More information on the magnitude of HGT operating in the human intestine came from the study of Zaneveld et al. (29). They revealed that enteric-adapted genomes are more comparable in gene content at a given evolutionary distance than non-gut genomes. Thus, common functional needs or magnified HGT causes similarities in genes within the gut compartment. Notably, niche specialization at short phylogenetic distances is also important in the mammalian intestine. More recently, a hypothesis that the animal gut is a melting pot for HGT has been summarized in two mini-reviews that surveyed HGT events in the mammalian gut and the role of HGT in the long-term adaptation of microbes to the intestinal milieu (30, 31). The authors concluded that the mammalian intestine is “a melting pot of genetic exchange, resulting in the large extent of HGT occurrence” (30).

Most of our knowledge of HGT has been obtained from the *in vitro* studies. Where the features are substantially different, especially in the gut, due to extremely dense and diverse microbiota. The plethora of functions include the suppression of settlement by pathobionts, degradation of dietary and *in situ*-produced components, production of nutritional factors, modulating and maintaining a functional mucosal immunity, supporting inter epithelial tight junction integrity, and contribution to intestinal epithelial homeostasis. Recently, evidence of increased DNA exchange among the Bacteroidales species within the human intestine has been reported (32). Genes that are extensively exchanged among these species encode proteins involved in fitness and multiple cycles-like alterations of gene expression. Fimbriae components may enhance attachment, utilization of new substrates increase the nutritional base, and secretion of antimicrobial molecules may confer a competitive advantage within the ecological niche. The genetic content of the “transferome” suggests that the gene transfer from the successfully adapted members of an ecosystem confers useful properties to the recipients, increasing their fitness and conferring them a competitive edge within the gut microbial ecosystem.

Humans and cultivated life stoke are the main consumers of antibiotics, thus enhancing HGT processes (24, 33–36). In fact, the gut microbiota represents a major habitat of antibiotic resistance genes (37–40), thus also called “gut resistome,” in the frame of the “gut mobilome” (41–43). Interestingly and somewhat alarmingly, high frequencies of HGT in infants’ meconium and early fecal samples have been recently reported (44). Antibiotic-susceptible commensal bacteria may acquire resistance to antibiotics *via* mutations in target genes or the acquirement of resistance genes by HGT, mainly by the transfer mediated by MGEs. MGE-mediated transfer of genetic cargo from one organism to another greatly contributes to the dissemination of antibiotic resistance genes, because it can take place between closely or non-related species and in diversified environments, including the animal and human gut (45–50).

Notably, the taxonomically distant prokaryotes of intestinal microbiome may share reservoir of closely related antimicrobial resistance genes (51). The role of bacteriophages, plasmids, conjugative transposons, and integrons in transfer of genes by pathogenic human enteric pathobionts and subsequent acquisition of pathogenicity also pointed to HGT as an important step in the expansion of virulence traits and antibiotic resistance (52–54). More so, existence of ancient and possibly, recent transfers of antibiotic resistance genes from antibiotic-producing actinobacteria to pathogenic proteobacteria was lately described (55).

The *in vivo* gut luminal milieu may encourage the transfer incidence and enhance the steady inheritance of MGEs, even in the lack of the antibiotic influence (56, 57). The soil microcosm exploration, represented by *Escherichia coli* as a donor with a genetically large conjugative plasmid RP4luc and the presence or absence of earthworms, supplied direct evidence that the gut passage is a prerequisite for a plasmid transfer to soil microbiota (58). Surprisingly, the plasmid transfer rates were even higher than can be achieved in filter mating experiments, suggesting that the HGT rhythm in nature could be higher than the laboratory assessed. If MGEs from soil can penetrate the earthworm gut, then they can lodge in the creature’s intestine that is next in the food chain, for example, moles and birds.

Additional example of the enhanced HGT under the gut environment has been demonstrated in ciliates (59). Ciliates are common in many aquatic ecosystems and in the rumen. Their food vacuoles are formed by phagocytosis and follow their path through the cell, thus, imitating a primitive enteric tract. Ciliates may enhance the rhythm of conjugal transfer between *E. coli* strains by two orders of magnitude, and the suggested pathway involved is the accretion of bacteria in vesicles allowing extensive HGT (59). This avenue may enhance the diffusion of antibiotic resistance in bacterial communities (60). The insect intestine can also be considered as a hot spot for HGT. For example, “the rates of conjugative plasmid transfer between *Salmonella enterica* Newport and *E. coli* in the intestine of the lesser mealworm beetle are by two orders of magnitude higher compared with filter mating” (61).

Conditions in the intestinal tract are highly expedient to HGT. The continuous flow of food, high density of microbiota, stable and optimal temperature, the formation of biofilm structures, and the vast diversity of enteric microbiota provide ideal conditions for HGT to occur in the human gut (62). Moreover, recent studies have disclosed the mechanisms of host–microbe molecular crosstalk that may enhance to the magnitude of HGT in the gut. It appears that the bacteria can perceive and react to host signals. For example, microbial sensing and responding to the level of host stress hormones is extensively reported (63–66). Even in the host–bacteria stress cross talks, a genetic material can be involved, expressed by the increased conjugative gene transfer between gut bacteria (67). It was shown *in vitro*, that “the physiological concentrations of norepinephrine enhanced the transfer of a conjugative plasmid from a clinical strain of *Salmonella* sp. to an *E. coli* recipient. Notably, the adrenergic receptor antagonists deprived the stimulatory effect of norepinephrine on conjugation. These signals of host stress may possibly affect HGT under the *in vivo* conditions as well” (5). In our recent work involving mice mono-associated with a human gut symbiont *Roseburia hominis*, we have discovered that a number of genes involved in HGT are upregulated in the bacterium in response to the intestinal environment (68). Finally, viral communities are abundant in the human intestine and viral sequences are transferred from bacterial cells to eukaryotic hosts (69). Not only phages are moving between microbes during antibiotic therapy but also whole phage communities are exchanged between human subjects during fecal microbial transplantation (69).

## HGT in Biofilms: Potential Applicability to Human Gut

Biofilm formation represents one of the main basic bacterial strategies for growth and survival in nature and disease (70). Biofilms are communities of microbes embedded in matrices composed of extracellular polymeric substance, and they were implicated for both the healthy and disease states of the host. Biofilm habitats are common in many biological ecosystems. The majority of microbiota found in natural, clinical, and industrial settings persist in association with surfaces and not in the planktonic state. They are usually found in many ecosystems including the teeth of humans and animals, and in the intestinal lumen (71).

The immune system recognizes many different bacterial patterns, but these components can be camouflaged in the biofilm mode of life. Transition from the planktonic to the biofilm-associated state induces bacterial production of small molecules, which can increase inflammation, induce cell death and necrosis and may potentially enhance posttranslational modification of naïve proteins to immunogenic ones, thus provoking undesirable immune reactions (72, 73). While planktonic cells are readily cleared, cells in biofilms are much less susceptible to clearance by neutrophils and macrophages. Moreover, in the presence of these host cells, biofilm formation is enhanced, and the components of the host immune system can be incorporated into the extracellular polymeric substance matrix (71). In particular, biofilm formation in the gut is facilitated by human secretory immunoglobulin A molecules (74). And the biofilm environment is well known for the HGT-promoting properties (5).

Cooperative phenotypes are important for the functioning of bacterial communities in many contexts, including syntrophy, linking *via* quorum sensing, biofilm formation, exchange of antibiotic resistance, and progress of polymicrobial infections. The human gut accommodates a dense and diverse microbial population critical to health, yet, cooperation within this important ecosystem, which has evolved over a long coevolutionary process, is poorly understood (75). Based on the above mutual, bidirectional, cooperative, and fine-tuned equilibrium between us and the microbiota, several questions may arise, such as, whether there is a risk associated with perturbation of this equilibrium, or, whether probiotics and the use of recombinant enzymes in foods may affect HGT in the human gut, or, if the changed microbial/microbial product profile could affect human health.

## Probiotics

The first person to bring the idea of colonization of the intestinal tract by advantageous microbes was Élie Metchnikoff, who observed, more than 100 years ago, that the largest percentage of centenarians live in Bulgaria. He related the phenomenon to the increased consumption of milk fermentation products, in particular Bulgarian yogurt. He forwarded the hypothesis that aging is caused by toxic bacteria in the gut, and he encouraged the use of Bulgarian yogurt and its principal component, *Lactobacillus delbrueckii* subsp. *bulgaricus*, to prevent this toxicity (76). The theory of aging did not hold but the encouraged use of lactic acid producing bacteria as helpful for health has grown enormously and, in fact, the global probiotics market extended to USD 27.9 billion in 2011 and is anticipated to reach USD 44.9 billion in 2018 (77).

The rostral definition of probiotics introduced by Lilly and Stillwell (78), however, had another meaning than that of the Metchnikoff’s, and it has been allocated to the “protozoa, in particular to the growth promotion of *Tetrahymena pyriformis* in response to a factor produced by *Colpidium campylum*. After several refinements, the definition has been assembled with the original idea of Metchnikoff,” and the actual definition of probiotics is “live microbial feed supplement which beneficially affects the host animal by improving its intestinal microbial balance” (79).

Frequently used probiotics include lactic acid producing bacteria, particularly lactobacilli, bifidobacteria, lactococci, and streptococci. Less often used are yeasts, bacilli, and non-pathogenic *E. coli* strains. The majority of probiotics, therefore, are facultative anaerobes, and their main effects are mediated through the secretion of lactate, and other short chain fatty acids that may inhibit the pathobionts and affect the communities of the enteric microbiome. Also, it is presumed that the intake of probiotics may modulate the immune system.

## HGT Potential of Probiotics

Probiotics and starter cultures have a generally regarded as safe status. The stature, however, had been acquired well before the recent safety concerns such as the carriage of antibiotic resistance genes on MGEs have been raised. From the safety standpoint, it is necessary to distinguish between the intrinsic resistance, which may constitute a normal physiology and metabolism (for example, inability of an antibiotic to enter the cell because of a particular cell wall structure or formation of a thick capsule) and the transferable antibiotic resistance genes (38). Phenotypic screening of probiotics in dietary supplements, for example, revealed a substantial level of antibiotic resistance, in particular, toward vancomycin, streptomycin, aztreonam, gentamicin, and/or ciprofloxacin antibiotics (80). Suggestions for risk assessment of antibiotic resistance in probiotic supplements obviously include broader genetic screening as well as the use of computational simulations, dynamic imaging, and functional genetics (81). Genes encoding virulence factors are also of concern, especially if they are located on MGEs and can be exchanged between the probiotic and commensal strains (82).

There is a growing body of literature on interspecies genetic exchange, underlining the importance of gene acquisition/loss within or between various probiotic strains. Examples of HGT among the probiotic strains have been reported for *Lactobacillus rhamnosus* (83), *Lactobacillus gasseri* (84), *Lactobacillus paracasei* (85), *Lactobacillus reuteri* (86–88), *Lactobacillus plantarum* (88), and some other probiotics. As mentioned earlier, the market for probiotics (and of course the consumption of them) is growing rapidly. Also, the gastrointestinal tract is the hot spot for the HGT events. Would the ingestion of probiotic cultures, which may act as donors or recipients, therefore increase the antibiotic resistance gene pool in the enteric ecosystem? Some authors suggest that there is a gene flux from Gram-positive cocci such as enterococci or streptococci to Gram-negative bacteria, with genes encoding for streptogramin resistance as an example (89). Being lactic acid producing bacteria, they contain plasmids containing genes conferring resistance to tetracycline, erythromycin, chloramphenicol, lincosamide, macrolides, streptomycin, and streptogramins (90).

*Leuconostoc* and *Pediococcus* species can serve as recipients for the broad host range antibiotic resistance plasmids from *Lactococcus* species (91). Conjugation transfer from enterococci to lactobacilli and lactococci can take place in the gastrointestinal tract of animals as well as *in vitro*; the transfer frequencies to lactobacilli, however, are pretty low (92). A recent systemic review has concluded that there is not enough evidence for the impact of probiotics on the stool microbiota composition in healthy human being (93). However, while the bacterial composition is not affected, the gene pool exchange *via* HGT between the probiotic and endogenous strains could happen. For example, the transfer of a tetracycline resistance gene from probiotic *L. reuteri* to bacteria in the human gut has been reported (94). To deal with this problem, Rosander et al. (95) removed antibiotic resistance gene-carrying plasmids from the commercial strain of *L. reuteri* ATCC 55730. Besides the antibiotic resistance genes, the danger of amplification of which is well recognized, other factors of concern could be toxins and virulence factors. Most recently, antibiotic resistance gene prevalence was described in the gut commensal bifidobacteria community, which is widely used in food processing and as probiotics (96). Notably, the acquisition of antibiotic resistance genes is age dependent, with a substantial increase during the first year of life.

Fittipaldi and others (97) reviewed more than 70 virulent factors in the zoonotic agent *Streptococcus suis*. Interestingly, the enzyme microbial transglutaminase, which is extensively used in the processed food industries (98–100), has been recently described as a virulence factor in this bacterium (101). Finally, the resistome present in probiotics is most probably underestimated and the double-edged sword effects of probiotics such as health benefits vs antibiotic resistance gene dissemination to the gut microbiome have to be carefully contemplated (102). The safety issues associated with the antibiotic resistance gene pool in probiotics have also been noted earlier (103, 104). Most recently, we explored the bidirectional effects of the human gut symbiont *R. hominis* in tissue models and in mono-associated mice (68). This interaction results in the concordant gene expression patterns at both sides. A set of genes considered to be important for bacterial–host colonization was induced in the bacterium. Even more interestingly, the host environment strongly induced genes involved in HGT, chemotaxis, and motility. The host responds by the enhanced expression of genes involved in innate immunity, gut barrier functions, and by increased Treg population expansion (68). Probiotics can potentially respond in a similar way, with the increased expression of genes involved in HGT, upon exposure to the gut environment.

## Transgenic Food Products

The topics of potential risks associated with the intake of genetically modified organisms or the so called “transgenic organism,” by humans and animals have been investigated in several of feeding trials. Interestingly, there is a shortage of support that DNA of transgenic plants, can be taken up by enteric microbiome or enter the organs other than the gastrointestinal lumen. “Neither tiny segments of transgenic DNA nor immunogenic fragments of transgenic protein were found in loin muscle samples from pigs fed a diet containing Roundup Ready soybean meal” (105). “Evaluation of the survival of transgenic plant DNA in the human gut deducted that gene transfer did not occur during the feeding experiment involving genetically modified soya” (106). No traces of endogenous soybean DNA could be traced in muscle samples of rats fed soybean meal from roundup ready or conventional soybeans (107). Likewise, no signs of transgenic DNA were traced in the milk of cows fed corn silage from an herbicide-tolerant genetically engineered products variety (108). Plasmid and genomic DNA from genetically modified plants were used in *in vitro* and *in vivo* transformation studies, but no detectable transfer of DNA was found (109). Attempts to detect DNA exchange from transgenic plants to bacteria in the gut of the tobacco hornworm (110) or bees also failed (111).

## Industrially Processed Food

There are a substantial number of microorganisms that are used in food production. It is estimated that about a quarter of all food production such as sausages, ham, cheese, and dairy products involves bacterial fermentation processes using lactic acid bacteria and other microbial and fungal strains. Here, the possibility of HGT among the bacteria used is more plausible. For example, when *Leuconostoc* and *Weissella* species, which are used as a mixed culture for aroma generation in traditional Italian and Spanish fermented cheese, were analyzed for antibiotic susceptibility, evidence for HGT was detected, demonstrating interspecies lateral gene transfer (48). In conjugation experiments performed both *in vitro* and in cheese, the transfer of erythromycin resistance between *Leuconostoc mesenteroides* and *Enterococcus faecalis* was detected.

While antibiotic resistant pathogens portray a straight threat to human and animal health due to difficulties of their uprooting (2), resistance among commensal bacteria constitutes an indirect hazard as being a reservoir of antibiotic resistance genes that can be shifted to pathogens *via* HGT. These reservoirs, therefore, represent a potential source for the dissemination of transmittable genes in bacterial ecosystems, including foodstuff. Food commensal microbes are a potential important avenue for HGT (112). For example, *Lactococcus lactis* that is associated with the Spanish traditional raw milk product was reported to transfer genetic material to lactococci and enterococci (113). Thus, food-borne commensal bacteria could be a potential source for the transfer of antimicrobial resistance genes, which is an important threat for public health (48, 114).

A recent study, which investigated samples from patients and healthy humans, farm animals and food, revealed a reduced dissemination of genes encoding extended-spectrum β-lactamase (ESBL)/pAmpC and plasmids bearing these genes from foods and farm animals to healthy humans and patients (115). Poultry and chicken meat represent a potential reservoir and a route for dissemination of these genes to humans. Although no evidence for the clonal spread of ESBL/pAmpC-producing *E. coli* from farm animals or foods to humans was found, ESBL/pAmpC-producing *E. coli* with same resistance genes and plasmids were present in farm animals, foods, and humans, suggesting HGT as a prevalent mechanism for the dissemination of antibiotic resistance genes.

Diet-driven convergence in gut microbiome functions has been investigated with 33 mammalian species and 18 humans (116). Microbiota adaptation to diet was reproducible across different mammalian lineages. Functional microbiota genes could be predicted from bacterial species assemblages, thus providing insights into the mechanisms driving the evolution of the enteric microbiome at the supra-organismal level. In the context of this review, the question is what was the contribution of horizontal gene exchange in the gut to the diet-derived convergence reported above? Redundancy in the repertoire of functional genes, for example, for complex carbohydrate utilization among various taxonomic groups suggests extensive exchange of these genes in the gut microcosm.

An entire different aspect of the industrial food production is food additives, which have been recently implicated as potential drivers of autoimmunity as affecting the integrity of intestinal epithelium tight junctions (98). Microbial transglutaminases, which are important for the survival of bacteria in nature, are extensively used in industrial food production as cross-linking agents of proteins, thus improving the texture and appearance of food products (73). They belong to the family of transglutaminases, which are functionally close to the endogenous tissue transglutaminases implicated as autoantigens in celiac disease. It has been suggested that microbial transglutaminases may be involved in the development of celiac disease (99). They have been demonstrated recently as immunogenic in celiac disease patients, representing a new marker that reflects the intestinal damage (100). In pathogens such as *S. suis*, the enzyme is a virulence factor, conferring antiphagocytic properties (101). It is not clear whether microbial transglutaminases in other bacteria may play a role in virulence. Given the extensive HGT events in the gut ecosystem and potential to enter human cells, it can participate in celiac disease progression (99) or in some other autoimmune diseases.

## Livestock and Companion Animals

The issue of possible zoonotic spread of antimicrobial-resistant bacteria and the corresponding genes is complex. Ewers and others (117) reviewed data available for *E. coli* isolates from livestock and companion animals. Most of these studies analyzed the chromosomal setting, with multilocus sequence typing, and the plasmid (episomal) ESBL/AmpC genes. In contrast to the diversity of episomal ESBL/AmpC types, isolates from human and animals mainly shared identical sequence types, suggesting gene transmission pathways of zoonotic bacteria, including multiresistant ESBL-producing *E. coli* or parallel microevolution. Another work from this group revealed that urban rats might be significant in regard to the human health because of high carriage rates of *E. coli* strains that have genotypes resembling those that circulate in human patients and thus can to be regarded as zoonotic (118). There is also direct evidence that the high carriage rate of “ESBL/AmpC-producing *E. coli* by poultry at broiler farms results in a high prevalence of ESBL/AmpC-producing *E. coli* in farmers” handling them (119).

Extended-spectrum cephalosporin-resistant Enterobacteriaceae (ESCRE) are found in humans and animals and in various environments. Circulation of these bacteria among the ecological compartments, therefore, is complex, due to multiple reservoirs and different transmission routes. Moreover, Enterobacteriaceae, including ESCRE, can be a part of the normal gut microbiota of healthy humans and animals, including dogs. Most recently, evidence for the household transfer of ESBL/AmpC-producing Enterobacteriaceae between humans and dogs has been reported (120). Of note, ESCRE are also found in food-producing animals worldwide and in meat products, potentially spreading from animals to humans through the food chain. ESCRE are also found in healthy and diseased companion animals such as dogs, and the potential zoonotic risk of this is emphasized (121).

But the risk is not limited only to dogs. Various antibiotic resistance genes and MGEs in the microbiota of many livestock and companion and wild animals have been described. One of the important factors contributing to their broad dissemination could be the selective pressure of antibiotics widely used in agriculture, especially in food-producing animals (36). A recent systematic review has tried to answer the question whether human extraintestinal *E. coli* infections resistant to expanded-spectrum cephalosporins arise from food-producing animals (122). Overall, there is the evidence that a proportion of human extraintestinal ESCR *E. coli* infections could exist, with poultry as a prime suspect. In contradiction to this, no evidence of clonal spread of ESBL/pAmpC-producing *E. coli* from farm animals or foods to humans has been found (115). However, ESBL/pAmpC-producing *E. coli* with identical genetic sequences and plasmids are present in farm animals, foods and humans thus suggesting a key role of HGT in circulation of ESBL/pAmpC among the microbiota of different ecological compartments. A recent large-scale analysis has revealed that the mobile resistome transfer network is shared between the human and animal gut microbiomes as well as by various human pathogens (49). Thus, the MGE-mediated transfer of antibiotic resistance genes is a more prevalent mechanism than the clonal dissemination of antibiotic resistance.

## Synthetic Biology Products

Synthetic biology tends to understand, reorganize, and control biological constituents to make functional units. The iterative process of designing and testing gene circuits has the potential to produce extensive valuable information into the mechanisms of the underlying functions of cells. It appears that, synthetic biology converges with disciplines such as systems biology and even classical cell biology, representing predictability to gene expression, cell biology and cellular signaling pathways. It can be simply summarized as “creating to understand.” This novel strategy uses the accumulating knowledge in the prokaryotic genetics to impact the eukaryote biological behavior (123).

Well known is the fact that the majority of microbes dwelling inside the human body are non-pathogenic and some of them can be turned, after appropriate engineering, into “smart” live therapies with defined capacities for the treatment of various morbid conditions. The use of engineered prokaryotic organisms to treat diverse human pathologies is constantly expanding. Some of diseases targeted are inflammatory bowel disease, autoimmune disorders, cancer, metabolic syndrome and obesity, neuropsychiatric disease, bacterial and viral infections as well as the development of vaccines against infectious diseases (124–126). In fact, synthetic biology strategies use microbial or viral reprogramming as human therapeutics, including novel means for strict bio containment. Another potential aspect of HGT is based on data demonstrating that DNA may be transferred between somatic cells *via* the incorporation of apoptotic bodies (127). The procedure allows the transfer of viral genes that have been merged with the genome in a receptor-independent fashion. Transferred DNA is multiplied and spreading inside daughter cells, in cell that have an inactivated DNA response which may change aberrant cell progression.

Based on the above, one can foresee several new therapeutic strategies, based on synthetic biology (126). “Living pills” are the engineered bacteria having a potential to deliver, enhance, or act as therapeutic modalities to treat various diseases (128). Such a pill, harboring the engineered microbe(s), with high enzymatic activity, high output of metabolic or proteomic product and easily deliverable, might be beneficial (73). Ongoing efforts are focused on adaptation of commensal bacteria for remodeling the gut ecosystem toward disease-treating condition (129). Engineered viruses can be adopted to selectively destroy pathogenic bacteria (130, 131). The synthetic biology can also represent a new avenue for HGT between bacteria, viruses, and somatic cells. We are not at this stage as yet, but the potential risks have to be carefully evaluated beforehand by regulatory bodies overseeing food safety such as the FDA, EFSA, and other national agencies at respective countries.

## HGT in Eukaryotes

The increasing body of evidence is accumulating on the HGT events in more complex eukaryotic organisms as well, which allowed them to acquire novel beneficial traits. In arthropods and nematodes, for example, the laterally acquired genes allowed to surpass plant defense mechanisms or broaden the nutritional base by acquiring the ability to degrade plant material as well as metabolizing a host-derived substrate (132–134). Traces of such lateral genetic transfer events can be found in genomes of many eukaryotes such as porifera, cnidaria, rotifers, nematodes, insects, arachnids, crustaceans, urochordates, and vertebrates (25, 135, 136). Transfer events among divergent species were mediated by various MGEs such as retroviruses, transposons and terminal-repeat retro transposons and preserved in the respective genomes.

Crisp and others (25) have recently described between ten and hundreds of potentially foreign genes that are expressed in primates, flies, nematodes, and humans. The majority of these genes are involved in metabolic pathways, suggesting that these laterally transferred genes contributed to the biochemical diversity during the early stage of invertebrate and vertebrate evolution. The authors suggested that a total of 145 human genes could be of bacterial origin, transferred to humans *via* HGT. Alignment of human genome against 53 vertebrate genomes has identified 1,467 human genome regions (2.6 M bases in total) as being more conserved with non-mammals compared with the majority of mammalian genomes (137). The authors concluded that HGT in the past might had a substantial impact on the human genome composition.

Several authors suggested that the interdomain gene transfer from bacteria and archaea into eukaryotes is more common than could be expected in the eukaryote and human genomes (137, 138). Acquisition of foreign genes *via* the mitochondrial or plastid or other organelle progenitors is not sufficient to explain this frequency and the weak-link model was proposed suggesting that the genetic transfer into multicellular eukaryotes happens during the unicellular or early developmental stages (139).

Other researchers have reservations regarding the magnitude of HGT to the human or other eukaryotic genomes (140–144). The arguments are as follows: the “foreign” DNA can represent a contamination; it can be a known mitochondrial or retro transposed gene; they represent gene loss or evolutionary rate variation; they can represent genes that evolved more rapidly in multiple lineages; and the allowance of a substantial error rate in the datasets with little statistical power. In fact, genetic contamination was reported as false signals of HGT as early as 2002 (145). A more recent controversy regarding the influx of prokaryotic genes to a eukaryotic genome is the tardigrade case (146). A substantial bacterial contamination could have contributed to the unusually high proportion of presumably HGT-acquired genes in this organism reported in the original publication. Due to the abovementioned disagreements and disputes, such studies should be subjected to close scrutiny and critical assessment, before concluding that the lateral genetic transfer represent a real biological event (141).

Besides the traces recorded in genomes, there are examples of viral and bacterial DNA incorporations into the human somatic genome that can be witnessed *in situ* (147). Integration of viral DNA into the host cell genome *via* HGT is a well-documented event in the human papilloma virus cancer development. Analysis of sequences from the Cancer Genome Atlas supports bacterial DNA integration into the nuclear chromosomes of adenocarcinoma stomach cells. Researchers from the same group have shown that cells from acute myeloid leukemia contain bacterial sequences that are present in the microbiome (148).

Based on the above information on HGT in eukaryotes (149) and among the other domains, the following is the hypothesis on the potential involvement of HGT in human chronic disease, autoimmune diseases being an example.

## Can HGT Play a Role in Autoimmune Disease?

Autoimmune diseases have both genetic and environmental components. While the genetic component can be investigated in a more or less straightforward manner by studying genetic variations associated with these diseases, the environmental component is much more difficult to decipher because of the involvement many variables and many combinations thereof. Besides, it is clear that the genetic component could not been changed substantially during the relatively short period of human evolution to explain the recent and dramatic rise of autoimmune diseases in human populations (150). Thus, the role of environmental factors in the onset of autoimmune diseases becomes increasingly recognized (151).

One of the most essential factors that can be considered as environmental, although it resides inside us, is gut microbiota. Its composition is largely driven by the lifestyle and diet, which have been dramatically transformed during the human history. In the history of human nutrition, there have been two major changes in the diet. The first included the carbohydrate-rich Neolithic diet, with the introduction of farming (~10,000 years ago), and the more recent second that involved the introduction of industrially processed food such as flour, sugar, and other refined products (beginning from ~1850) (152–154). The view is emerging that these changes resulted in a substantial loss of gut bacterial diversity (155, 156). Presumably, the functional diversity is affected as well, thus, changing the interface of host–microbe interaction, with less appropriate functional and signaling properties of the microbiota. The resulting dysbiotic configuration potentially could make a significant contribution to the landscape of contemporary diseases, which have neither a substantial genetic component nor infectious nature (157–159). The factor contributing to the rise of these diseases could be the contemporary lifestyle with limited microbial exposure. Excessively hygienic conditions may compromise the establishment of normal microbiota and negates the immune benefits associated with it (160, 161).

As mentioned before, contemporary intensive agricultural systems with the widespread use of herbicides, insecticides, fungicides, fumigants, desiccants, harvest aids, antimicrobials, growth regulators, metals, and many other substances, are evident. The long-term health effects of the residual concentrations of these chemicals in our food are largely unknown. It has been hypothesized, for example, that the obesity epidemic in the United States may be partly due to the mass exposure of citizens to food containing low-residue antimicrobial agents (162). The resulting dysbiosis may predispose to obesity and, potentially, other diseases.

In this review, we discussed how the HGT mechanisms and processes that have been selected during the previous long-term human–microbiota coevolution may interact with the new reality potentially leading to dysbiotic conditions and even disease (98, 99, 163, 164). HGT in the human gut is intense, and the material for genetic exchange can come from various sources such as microorganisms, viruses, ingested probiotics, fermented and processed food products or industrial additives, plants, livestock, proximity to companion animals, and synthetic biology products. In the human microbiome, the microbial enzymatic machinery, especially involved in posttranslational activity, may modify naïve proteins to immunogenic ones (72, 73, 99, 165, 166). Bacterial or viral infections (167–170) could also impact the fine tuning of the intestinal homeostasis. One of the targets affected by these microbial activities is the intestinal barrier mechanism represented by the very evolutionary conserved inter-enterocyte tight junction. The leaky gut syndrome is central to the development of various autoimmune diseases, where the luminal content can enter the circulation and initiate systemic inflammatory responses (171). The inflammation that is not properly resolved may lead to chronic inflammatory conditions including autoimmune diseases.

We propose a hypothesis here that, due to extensive inflow of genetic material to the intestine and high intensity of HGT in the gut, a substantial proportion of these genes may be expressed. This may result in the production of neo-protein or neo-peptide formation that imitates self-ones. This molecular mimicry or epitope spreading are well known pathways in autoimmunity inductions. The leaky gut condition results in translocation of the luminal content thus enhancing the inflammatory cascade. If the inflammation is not resolved properly, it is transformed into the self-sustainable chronic inflammation and tissue damage, with a risk of exposure of own antigens to the immune system. The resultant autoantibodies may exacerbate the situation further, with tissue damage and amplification of inflammatory cascades. The current hypothesis is shared, partially, with the hypothesis suggested by Robinson and others (8), on the role of the bacteria–animal HGT in carcinogenesis. It should be emphasized here that this is a hypothesis and, as such, has to be verified in future studies. It is hoped that the hypothesis will encourage the scientific community to explore the relevance of the gut HGT to the onset of chronic human diseases, including, autoimmune diseases.

## Conclusion

As a product of long-term cospeciation with the host (172), commensal gut microbiota had evolved to perform many functions such as contributing to host nutrition *via* degrading dietary components inaccessible by host and synthetizing vitamins, processing and detoxifying xenobiotics, regulating host development and metabolism, conferring colonization resistance toward pathogens, and shaping and maintaining mucosal and systemic immunity. These functions are governed by the specific sets of genes, for which the gut microbiome is particularly enriched compared with other microbial ecosystems. The putative gut-specific genes include those involved in adhesion to the host proteins, in harvesting sugars of the glob series glycolipids, and in degradation of dietary or host-derived complex sugars and glycans (173). The biggest proportion of genes in the minimal gut metagenome (about 5%), however, codes for (pro) phage-related proteins, implying the important role played by these MGEs in the human gut ecosystem.

Other processes associated with changes in lifestyle potentially contributing to the increase in HGT rates in the gut may include the use of antibiotics, the probiotics ingested, food products processed *via* fermentation, and proximity to livestock and companion animals (7, 38, 126, 174). The best-known laterally transferred genes that had already caused serious concerns are those conferring resistance to antibiotics. Their rapid emergence and broad dissemination among taxonomically diverse species within a relatively short period of time demonstrated the extreme adaptability of bacteria (18), which is based on their naturally possessed genetic engineering tools such as MGEs (5).

Besides the responses against the selective pressure of antibiotics, there are other examples of adaptive traits acquired by gut bacteria *via* HGT, for example, in response to dietary habits. Japanese diet is traditionally reach in seaweeds and the gut metagenome of the Japanese contain porphyranases and agarases that are absent in the metagenomes of other populations (175). Presumably, in these populations, the enzymes were acquired by gut microbiota (in particular, *Bacteroides plebeius*) from marine bacteria through HGT, and they became completely functional in the new ecosystem to aid the host in digesting these dietary compounds (176).

**Figure 1:**
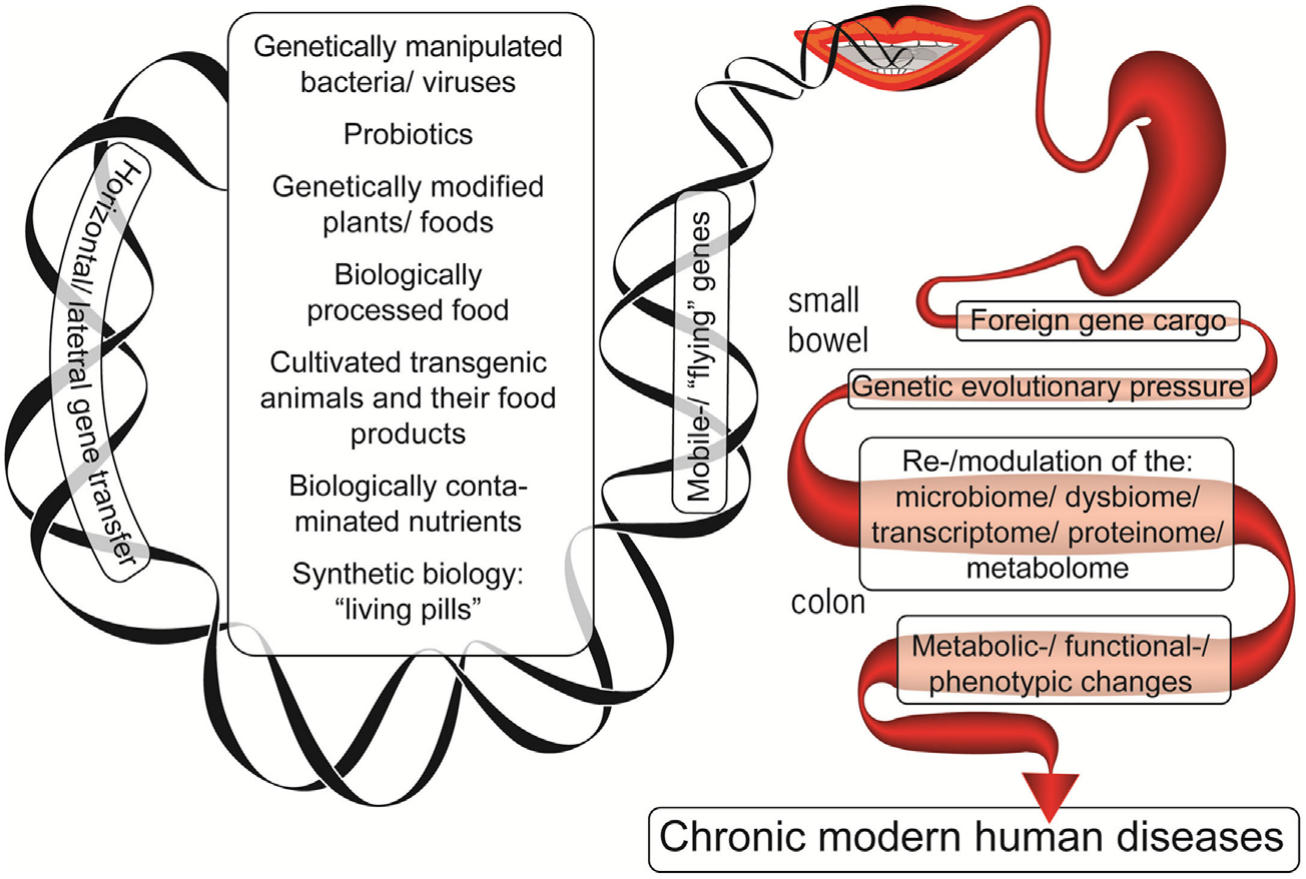
The foreign genetic cargo that environmental factors can deliver by horizontal gene transfer to the enteric microbiome, resulting in chronic human diseases.

The example above demonstrates how the microbiome may acquire genes from transient bacteria and successfully integrate them. In the current lifestyle, the microbiome becomes even more exposed to a gene inflow originating from the contemporary production of livestock, plants, industrially processed food, probiotics, and food supplements (Figure 1). Despite the fact that direct causal relationship between all those consumed constituents and chronic human diseases’ induction is lacking, the present review highlights the potential risks associated with the modification of the evolutionary established stability of our microbiomes. Given the extensive HGT processes in the gut, assimilation of these incoming genes may affect the previously established and fine-tuned host–microbe interaction. Thus, these two factors of the contemporary lifestyle and dietary habits, i.e., a lessened exposure to natural microbiota and the increasing exposure to the previously un-encountered ones, may affect the host–microbe crosstalk and compromise the host health. Indeed, we currently witness a tremendous increase in human diseases that have a substantial gut microbiome component.

The gut microbiota is very dynamic and, on the evolutionary scale, responds almost immediately, *via* HGT mechanisms, to various selective pressures imposed, which is well exemplified by the recent history of antibiotic use, which resulted in the widespread antibiotic resistance (177). One of the current functions of the gut mobilome under continuous antibiotic selective pressure, as serving as the antibiotic resistance gene reservoir, is well established (178). Concomitantly with selection for antibiotic resistance *per se*, this pressure may result in the change of the virulome profile as well. Although the relationship between antibiotic resistance and virulence is complex (22), there is a number of examples how these two traits evolved simultaneously *via* the common mechanisms (179). It is not fully understood, however, what is the impact of subtler selective pressures that is applied on gut microbiota through the use of probiotics, fermented food, food with the presence xenobiotics and antibiotics, and various food supplements. The effect of genetic exchange with the microbiota of livestock and pet and companion animals is also not well understood. The mobilome-driven genome diversification in the gut is very intense (180) and, undoubtedly, the selective pressure imposed by us has already resulted in the selection of certain genes and genotypes in the microbiome. In the light of the increasing number of diseases and conditions having inflammatory and autoimmune components linked to gut microbiota (171), we have to admit that the best microbiome configuration has not been selected by our contemporary lifestyle and dietary habits. Understanding and then correcting this imbalance may greatly contribute to our health and well-being. Notably, at least in animal model, autoimmunity can be tackled by gene transfer to promote immune tolerance (181, 182). We hope that the hypothesis proposed here will encourage the medical and scientific communities to evaluate the risks associated with HGT events in the human gut.

## Ethics Statement

The study does not involve any human or animal subjects.

## Author Contributions

AL and RA screened literature, analyzed the data, and wrote the manuscript. TM designed, edited, and revised critically the manuscript.

## Conflict of Interest Statement

The authors declare that the research was conducted in the absence of any commercial or financial relationships that could be construed as a potential conflict of interest.

## Abbreviations

HGT, horizontal gene transfer; MGEs, mobile genetic elements.
